# Single-Cell Analysis 2.0

**DOI:** 10.3390/cells12010154

**Published:** 2022-12-30

**Authors:** Tuhin Subhra Santra, Fan-Gang Tseng

**Affiliations:** 1 Department of Engineering Design, Indian Institute of Technology Madras, Chennai 600036, India; 2 Department of Engineering and System Science, National Tsing Hua University, Hsinchu 30013, Taiwan

In 1665, Robert Hooke published his revolutionary book Micrographia [1], where he wrote about his observation of box-shaped structures under a microscope. It was for the first time that he gave these little structures the name ‘cells’. After almost 170 years, when the classical cell theory was developed, it was concluded that these cells are in fact the fundamental functional and structural units of life. Subsequent biological research, particularly in the last hundred years, has resulted in significant discoveries about these cells. Today, it is well known that an individual cell weighs around a few nanograms and has a volume of around 1 pL [2]. We are also familiar with their subcellular composition, method of intracellular uptake, mechanisms of cellular proliferation, and differentiation, etc. However, despite these developments, there are many aspects of cellular functioning and behaviour that are yet to be comprehensively understood. Even as recently as 2019, the Nobel Prize in Physiology or Medicine was awarded for discovering the sensing abilities of cells for changing oxygen availability [3]–a fundamental characteristic of a cell.

A major requirement of biological research is to understand the cellular phenomena at the single-cell level. Despite similar compositions, individual cells are heterogeneous, and a bulk analysis of a cluster of cells provides only average information about the target characteristic. The heterogeneous nature of cells finds critical relevance in cancer studies, such as in the tumor microenvironment, where different layers of the tumor exhibit varying genotypic and phenotypic characteristics [4]. Again, the initiation of tumor metastasis can be conducted by a few cancer cells, which makes it very essential to quantify the developments at high spatial resolutions [5]. Heterogenic properties also hinder the development of accurate disease models and affect drug resistance studies [4,6]. Consequently, single-cell analysis is essential for understanding various cellular features, such as intercellular signalling, intracellular molecular processes, cell mechanical properties, spatio-temporal transformations, etc. Single-cell analysis also facilitates different omics analyses, such as proteomics, transcriptomics, metabolomics, etc., which can unveil critical information about cellular mutations [7]. 

With the advent of micro/nanotechnology, our ability to manipulate activities at the micro/nanoscale has enhanced rapidly. Significant developments in microscale imaging and high precision micro/nanofabrication over the past two decades have enabled researchers to have the capability of better understanding and visualising smaller scales. Today, the fabrication of microfluidic devices, nanostructures, etc. is easier and more economic than ever before. Single-cell studies using these micro/nanodevices find critical applications in the fields of cellular sensing, intracellular delivery, and disease analysis [8,9,10,11], leading to a wide variety of research on areas such as disease progression, regenerative medicine, and cancer therapy [2,6,12,13,14]. Consequently, the area of single-cell analysis has earned increased popularity in the past two decades [4,7,13,15,16,17,18,19,20,21]. In our previous special issue on Single-Cell Analysis [20] itself, we highlighted the significant rise in single-cell-related publications in the past two decades [7]. In continuation with that trend, the last two years has only witnessed an ever-increasing rise in single-cell research publications according to the Scopus Index and Web of Science, shown in Figure 1a,b. Within the areas that have been established, the greatest interest has been the understanding of molecular and cellular biology using single-cell-assisted studies. Other popular areas include immunology studies and stem cell research.

This particular Special Issue of *Cells*, titled, ‘Single-Cell Analysis 2.0’, contains 10 technical articles and two tutorial reviews. It is a compilation of a variety of studies, such as single-cell RNA sequencing, single-cell genomics, transcriptomics, intratumoral heterogeneity analysis, modulatory effects in mitochondrial membrane potential, reactive oxygen species (ROS), vesicle transport in single living human adipose mesenchymal stem cells (hADSCs), near-infrared (NIR) laser effect on mitochondrial dynamics, cell progeny of single pallial GFAP-expressing progenitors, single red blood cell analysis, surface charge distribution of single-cell, single-cell RT-qPCR, single-cell adhesion force kinetics, etc. Regarding the former, for example, Gao et al. [22] directed comparative single-cell genomics analysis using a 10× single-cell platform and revealed the single-cell resolution transcriptomic atlas of hematopoietic stem and progenitor cells (HSPCs) of humans and mice using a canonical correlation analysis (CCA) computational strategy. The study includes a total analysis of 32,805 single-cells with 15,245 single hCD34^+^ stem/progenitor cells and 17,560 linage^−^mCD117^+^ cells. The group used canonical correlation analysis (CCA) methods, such as Monocle, to examine the trajectories of hematopoietic differentiation, as well as SCENIC to analyze gene networks underlying hematopoiesis. This study interestingly found that the hematopoietic stem and progenitor cell (HSPC) compartments in the two species were composed of populations characterized by the similar sets of homologous genes. Additionally, the hematopoietic lineages and transcriptional profiling in hematopoiesis are well conserved between humans and mice. Finally, the study revealed the significant evolutionary similarity in the human and mouse hematopoietic systems at the single-cell level.

Lin et al. [23] carried out in-depth integrated canonical correlation analyses and revealed the subtle differential gene expressions (DGE) between in vitro derived totipotent blastomere-like cells (TBLCs) and in vivo mouse early developmental cell stages. The group interrogated the true totipotent nature of TBLCs and addressed it via single-cell RNA seq analysis between TBLCs and cells from early mouse embryonic developmental stages. Interestingly, the group revealed a subpopulation “Cluster 3” within the TBLCs population via the DGE analysis and found a high-level expression of the totipotent-related genes *Zscan4s* and displayed transcriptomic features similar to mouse two-cell stage embryonic cells. Therefore, this study resulted in the identification of a new experimental model in the area of stem cell biology, namely, ‘cluster 3’, as a subpopulation of TBLCs that could be molecularly defined as near totipotent cells.

Walbech et al. [24] investigated the simplification of autoencoders to model the single-cell mRNA-seq data across datasets and found that through a specialized autoencoder, biologically relevant data could be retrieved. Generally, one can extract biological modules, denoise, and classify data correctly from a trained autoencoder on a different dataset and with different cells (a foreign model). Thus, the authors demonstrated the use of trained interpretable autoencoders and saliency mapping to deconvolute biological signals encoded within scRNA-models [24]. This study highlighted that a skilled autoencoder could be diligently employed to interpret the new sets of unpublished data and, thus, epitomize a significant biological concept featuring a substantial biological pathway, respectively. Potts et al. [25], for the first time, utilized data from single-cell RNA sequencing (scRNAseq) to demonstrate that reading similar sets of scRNAseq can yield varying results when aligned to dissimilar genome assemblies. This generates dissimilarities in matrix dimension, gene expression patterns, and identifying cell-types.

In other studies, Ouyang et al. [26] proposed a simplistic technique for mapping the cell surface charge by utilising electrostatic, cell–nanoparticle (fluorescent) interactions. Their approach successfully evaluated the average value of the zeta potentials for two different cell types, which closely matched the values commonly obtained using electrophoretic light scattering. Again, Park et al. [27] performed a cellular morphological characterization by using an ultra-high throughput holographic cytometry method. The platform uses single-cell images to study cell phenotypes by gathering phase images from an extensive number of cells. Torab et al. [28] studied how different molecular subtypes interact within bladder microtumors. Their results indicate that heterogeneous microtumors (comprising of multiple molecular subtypes) are more invasive than individual cells, despite both cells not being individually invasive. Ojalvo-Sanz et al. [29] employed a genetic lineage-tracing method to target progenitor cells in lower cortical layers of embryonic mice brains and tracked their adult glial progeny. Their results emphasized the heterogeneity of pallial E14 neural progenitors with regards to the type of glial cell they produce and the clonal size of the progeny. Gbetuwa et al. [30] studied the effect of near infrared radiation (NIR) on the nucleus and cytoplasm of single-cells. Their results indicate that nuclear irradiation promotes mitochondrial fission, which is otherwise not observed with direct mitochondrial exposure. Pan et al. [31] also performed a single-cell NIR irradiation to investigate the effect of photobiomodulation in regulating properties, such as mitochondrial membrane potential, reactive oxygen species production, and vesicle transport. Their studies indicated an increase in the value of these cellular properties, which may hold great significance for different cellular responses, such as migration, differentiation, etc.

This particular issue also consists of two independent tutorial reviews. Shinde et al. [32] elaborate on the different methodologies used for measuring cellular and molecular adhesion forces. They discuss the significance of analysing cellular adhesion mechanisms to enable better quantification of the cellular mechanical properties. They also review the recent technological developments for performing single-cell adhesion studies. Zucha et al. [33] provide a comprehensive overview of the single-cell reverse transcription quantitative PCR (scRT-qPCR) method. They discuss the advantages and importance of scRT-qPCR as compared to other single-cell collection methods. The authors highlight the key factors that influence the performance of the method, such as preamplification, primer design, data processing steps, etc. They also provide theoretical recommendations and practical guidelines to facilitate the implementation of the method.

In conclusion, we expect that this second issue of Single-Cell Analysis 2.0 will provide readers and researchers with fresh insight into the area of single-cell analysis. Biological cells are extremely complex entities, and there are still many mysterious corners waiting to be discovered. We hope that these articles encourage new questions and consequently help the scientific community to make exciting findings about cellular behaviour, dynamics, therapy, and their analysis.

## Figures and Tables

**Figure 1:**
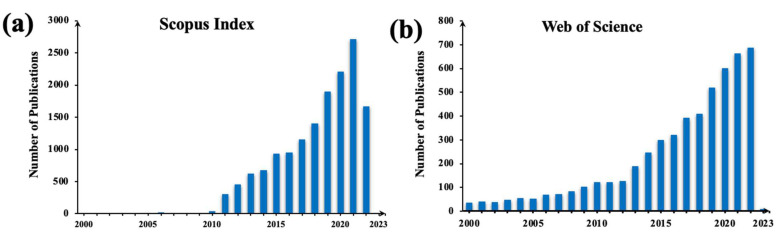
Year wise single-cell related article publications in the Scopus Index and Web of Science until 20th December 2022. (**a**) The data are adopted from Scopus Index articles (**b**) The data are adopted from Web of Science articles.
